# Pandemic Communities of Fate and Care Penalties Among Community Outreach Workers in California's Central Valley

**DOI:** 10.1111/1467-9566.70080

**Published:** 2025-08-29

**Authors:** Meredith Van Natta, Andrés Arias, Maria Andrea Escobar

**Affiliations:** ^1^ Department of Sociology University of California – Merced Merced California USA; ^2^ Department of Sociology & Anthropology Albright College Reading Pennsylvania USA

**Keywords:** care penalty, community of fate, community outreach workers, immigrant health, *promotoras*

## Abstract

Existing scholarship has examined community outreach workers' vital role as an essential, effective public health workforce in underserved communities. Less attention has been paid to how such workers have experienced this rhetorically praised yet materially undervalued labour in the context of the COVID‐19 pandemic. Drawing on interviews with 43 community outreach workers who facilitated health and well‐being services for immigrant communities in California’s Central Valley, we find that the pandemic created a unique ‘community of fate’ among community outreach workers in the region. This community of fate, in turn, exacerbated an existing care penalty rooted in the gendered, racialised and legally stratified nature of their labour. Taking both a micro‐ and macro‐level analytical approach, we argue that the symbolic value of this ‘essential’ labour—often expressed in terms of a calling, vocation or destiny—intensified its material devaluation despite the importance of their work in mitigating pandemic harms.

## 1 Introduction

In the United States, Latin American immigrant communities disproportionately bore the health and economic impacts of the COVID‐19 pandemic (Macias Gil et al. 2020; Garcia et al. 2021; Olayo‐Méndez et al. 2021). Federal exclusions from various public safety‐net programmes exacerbated this situation for many noncitizens and immigrant families (Makhlouf and Sandhu 2020; Parmet 2021). This was especially true in the rural United States, where health care and other social service institutions are few and far between (Grogan et al. 2021; Young et al. 2022). In such spaces, community‐based outreach workers constitute an essential resource for the excluded and under‐resourced communities of which they are a part—a role that became even more vital as the pandemic unfolded.

Scholars have emphasised the importance of community outreach workers—including *promotores* (outreach workers who typically focus on health needs), health navigators and certified enrolment counsellors—in facilitating health and well‐being services for communities in need (Lewin et al. 2005; Pérez and Martinez 2008; Burke et al. 2020). Scholars have noted, however, that although this outreach workforce often provides substantial benefits for communities and health systems, workers' wages and/or nonpecuniary benefits seldom correspond to the impact of their work (e.g., Maes 2015; Closser et al. 2019). Instead, as Closser et al. (2019) note, community health workers—predominantly rural women—'are frequently disempowered staff and the bottom of health bureaucracies, facing severe restrictions on their ability to advocate for themselves or for the needs of their communities’ (Closser et al. 2019, 300). Such scholarship underscores the absence of comprehensive policies to ensure outreach workers' fair pay, career advancement opportunities and meaningful representation in policymaking decisions (Closser et al. 2019, 304).

In the United States, the 2010 Affordable Care Act included provisions for expanding a community health outreach workforce, and research in the intervening decade‐plus has underscored such workers' effectiveness in health care and social welfare brokering (Morrissey 2011; Kim et al. 2016; Berini et al. 2022). This is especially true in immigrant communities, which face steep barriers to equitable and adequate care (Findley and Matos 2015; Islam et al. 2017; Hernandez‐Salinas et al. 2023). As existing scholarship on community health workers demonstrates, however, this impressive effectiveness often contrasts with community outreach workers' actual valuation, as they experience relatively low wages and limited professional recognition (Gutiérrez et al. 2021; Logan 2021). Building on our previous work (Van Natta et al. 2023) identifying specific pathways to this undervaluation—including organisations' inability to formally employ undocumented workers and the temporally limited availability of ‘emergency’ public COVID‐19 public funding—we demonstrate how the pandemic intensified this longstanding material‐discursive misalignment.

In this study, we ask how community outreach workers in a largely rural region of California experienced undertaking vital labour under precarious, unprecedented conditions. Despite their location within the world's fourth‐largest economy (Newsom 2025) and relatively intensive statewide safety‐net investment, Central Valley residents have suffered from longstanding state neglect via structural violence that fuels ‘syndemics’ (or synergistic epidemics) (Burke et al. 2025; Singer 2009). According to the California Budget & Policy Center, ‘California's vast wealth contrasts sharply with deep income inequality, leaving over a quarter of residents unable to meet basic needs without safety net support’ (Saucedo and Nair 2025). Many of these residents live in the Central Valley (Finocchio and Paci 2020).

Viewing Central Valley community outreach workers' experiences through the lenses of *community of fate* and the *care penalty*, we explore both the meaning they expressed regarding this work and the material conditions of frontline care work amid a protracted crisis. We argue that although participants often articulated their work in terms of labour done from the heart on behalf of beloved communities, their convergence as a ‘community of fate’ exacerbated the material undervaluation of vital outreach work as gendered, racialised and legally stratified labour through a ‘care penalty’ (Folbre et al. 2021). Such care penalties happen when the symbolic value of one's work misaligns with its economic and professional benefits. We demonstrate how a pandemic ‘community of fate’ among California Central Valley community outreach workers intensified a broader political economy of care work that is morally—but not materially—valued.

We first conceptualise community outreach workers as a ‘community of fate’ (Baehr 2005) and then explore how this compounds a care penalty for some of California's most vital but overlooked ‘essential’ workers. The term ‘essential’ here has two somewhat opposing meanings. During the early years of the pandemic, ‘essential’ workers were those whose labour had to continue uninterrupted for the sake of everyone else. Beyond medical personnel, this also included workers in agriculture, food processing and service, janitorial/hospitality, warehousing and manufacturing (UC Merced Community and Labour Centre 2021). The phrase ‘essential’ worker, however, could also describe someone whose work defines their essence. By engaging the notion of the community of fate, we demonstrate how this alternative idea of essential labour intersects with the ‘moral density’—or deep sense of a collective, socially entrenched problem that extends to the community level—of care work. This, in turn, rationalises a care penalty for community outreach workers, one that the pandemic compounded through outreach workers' community of fate.

### 1.1 ‘Communities of Fate’

We draw upon Baehr's reconceptualisation of Weber’s (2019 [1922]) ‘communities of fate’ to describe ‘a process of group formation under extreme duress’ (Baehr 2005, 181). Baehr emphasises that these communities reflect ‘a pattern of temporary social cohesion arising from a mass emergency or “disaster”’, which are unique precisely because they ‘*come into being* as a result of stress and crisis…’ (Baehr 2005, 181, emphasis in original). Like other scholars who apply this concept to pandemics (e.g., Baehr 2005; Montgomery et al. 2021), we engage this analytical lens towards a particular social group: community outreach workers in California's Central Valley during the COVID‐19 pandemic. We examine how these workers constituted a ‘community of fate’ and investigate its material consequences for such workers via their gendered, racialised and legally stratified social position.

Although scholars have taken up the notion of ‘communities of fate’ primarily in relation to labour organising, financial systems and transnational responses to large‐scale existential threats such as terrorism and climate change (e.g., Plankey‐Videla 2006; Beck and Grande 2010; Mitchell 2022), we focus on recent sociological applications in pandemic contexts. Such scholarship draws on Weberian interpretations of ‘community of fate’ that consider the subjective meaning of collective identity related to shared potential futures among similarly situated individuals (Baehr 2008, 119, citing Weber’s *Economy and Society* [1922]; Mitchell 2022, 177–8). Examples include Baehr's (2005) study of the 2003 severe acute respiratory syndrome (SARS) pandemic in Hong Kong, Figuié’s (2013) analysis of avian flu (H5N1) outbreaks in Vietnam from 2003 to 2007 and Montgomery et al.’s (2021) examination of critical care workers in England's National Health Service during the COVID‐19 pandemic.

To constitute a ‘community of fate’, Baehr proposes seven classificatory criteria (Baehr 2005, 184–91). These criteria are as follows: *danger recognition* (perceiving a certain, immediate threat that demands urgent action); *moral density* (a profound sense of a collective, socially entangled problem that extends beyond the family); *trial* (grasping the situation as not only an acute, but also sustained, shock); *closure* (realising that they have nowhere to go and share a common fate with those who are also trapped around them); *material and organisational resources* (possessing some tools and strategies to fight the menace facing them); *axis of convergence* (a shared language and/or rhetoric and ‘sense of social cohesion’—especially co‐ethnic ties) and *social ritual* (e.g., donning masks and personal protective equipment in public). Below, we provide examples of these criteria to demonstrate how community outreach workers formed a unique ‘community of fate’ during the COVID‐19 pandemic. We then situate these perspectives within the broader political economy of care work. By taking both a micro‐ and macro‐level approach, we argue that the existence of this unique community of fate exacerbated the material devaluation of care workers, thereby multiplying existing care penalties.

### 1.2 ‘Care Penalty’

Scholarship on ‘care work’—broadly defined not only as unpaid labour in the home, but also as ‘care’ professions such as child and elder care, nursing and teaching—emphasises persistent, intersecting gendered and racialised pay gaps rooted in patriarchy, White supremacy and colonialism (Parreñas 2001; England et al. 2002; Duffy 2011; Romero and Pérez 2016). More recent scholarship highlighting care workers' vital yet often precarious labour during the COVID‐19 pandemic suggests enduring contradictions between the value of this work for community health and the limited investment such professions experience (Logan and Castañeda 2020; Peretz et al. 2020; Folbre et al. 2021; Masquillier and Cosaert 2022).

Gender scholars have long emphasised the gendered and racialised nature of care work and examined possible mechanisms for its devaluation. In earlier reviews of the scholarship, England and Folbre (1999) and England (2005) describe various explanations for this ‘care penalty’, which include the devalued association of care work with women (especially women of colour), care work as a public good or intrinsic amenity that cannot be priced, and/or emotionally extractive and commodifying dynamics of care work in neoliberal markets. Likewise, Johnson (2015) examines how care work motivated by ‘naturally’ feminine inclinations towards altruism, empathy and voluntarism may make care workers ‘more prepared to accept a lower wage’ and/or ‘give of themselves voluntarily’ by accepting ‘the moral currency of emotion’ in lieu of financial remuneration (Johnson 2015, 117–119).

Additional historical and empirical studies have examined the interlocking dynamics of gendered and racialised care work, highlighting how women of colour—especially immigrants from the Global South—disproportionately bear this multifaceted devaluation through their over‐representation in low‐wage care (or no‐wage) professions (Glenn 1992; Dodson and Zincavage 2007; Hondagneu‐Sotelo 2007). Indeed, with respect to outreach work specifically, many programmes construct ‘workers’ explicitly as volunteers rather than a paid workforce on the premise that remunerating care work would undermine its intrinsically priceless nature and purely altruistic motivation. Such framing allows states and institutions to praise the positive impact of outreach work in communities while avoiding ‘valuing [community outreach workers’] labour by providing them with financial remuneration that might improve their own economic and social lives' (Maes 2015, 99; see also Graeber 2018).

These factors suggest the potential disempowerment of care workers, which extends beyond a pay penalty itself to a lack of control over exploitative and/or unsafe working conditions, ‘which is intensified when workers cannot walk off the job or go on strike without harming those they care for’ (Folbre et al. 2021, 184). Such insights are especially relevant to care work during the COVID‐19 pandemic. Like much of the rhetoric praising ‘essential’ workers during the pandemic, the discursive elevation of community outreach workers often misaligns with their material valuation—a fact that the pandemic intensified. Beyond the disproportionate physical and mental health impacts that public health workers experienced during the pandemic (e.g., Bryant‐Genevier et al. 2021; Recto et al. 2023), we demonstrate the exacerbation of a ‘care penalty’ via a ‘disjuncture between the social value of work and its private, pecuniary award’ (Folbre et al. 2021, 174). As Folbre et al. argue, the ‘distinctive features of care work—intrinsic motivation, emotional skills, team production and positive spillover effects’ often belie the fact that ‘essential’ workers in care services earn less than other similarly ‘essential’ workers (in law enforcement, waste services, transportation, agriculture and so on), illustrating ‘a pattern with especially costly consequences for women’ (Folbre et al. 2021, 174).

This observation underscores the tension between the ‘essence’ of care work as quasi‐altruistic, emotionally meaningful labour and the economic necessity of ‘essential’ work that is rhetorically praised yet materially undervalued. As emerging scholarship explores how COVID‐19 has reified the intersectional inequalities that sustain care work's undervaluation (e.g., Al‐Ali 2020; Ozkazanc‐Pan and Pullen 2021), we emphasise how the community outreach workforce, largely composed of women from immigrant families, is often motivated from the heart but materially constrained by intersecting gendered, racialised and legal status stratifications. We identify some limitations of existing community of fate frameworks to consider power and structural inequalities, arguing that the emergence of a pandemic community of fate of Central Valley outreach workers compounded the undervaluation of their work.

## 2 Data and Methods

We conducted semi‐structured interviews with community outreach workers who facilitated health and well‐being services on behalf of immigrant communities in California’s Central Valley. Unlike other California regions with relatively robust and immigrant‐inclusive safety‐net programmes, the Central Valley includes many rural, agricultural communities, where immigrant‐supportive safety‐net infrastructure is often absent. Thus, in a region where approximately 45% of households live below 200% of the federal poverty level (compared to 30% in California and 29% in the United States), and with the state’s greatest share of workers employed in jobs rated highest risk for COVID‐19 transmission, immigrant families experienced especially profound pandemic health and economic disruptions (Joynt 2024; UC Merced Community and Labour Centre 2021; Kaiser Family Foundation 2025). Moreover, once COVID testing and vaccines became available, the Central Valley remained far behind more resourced coastal regions in vaccine uptake (California Department of Health and Human Services 2023). These conditions subjected Central Valley immigrant families to excessive health and socioeconomic impacts—especially as federal restrictions prohibited many noncitizens and mixed‐status families from receiving pandemic relief (Makhlouf and Sandhu 2020; Parmet 2021).

Within this challenging context, study participants carried out their vital outreach work. The workers we spoke with fulfilled ‘frontline’ outreach roles related to various social determinants of health, including coordinating COVID‐19 education, testing and vaccination; facilitating nutrition, housing and unemployment assistance; and navigating complex state and federal eligibility criteria for safety‐net resources. Between October 2021 and June 2022, our team conducted Zoom and telephone interviews with 43 of these community outreach workers. Prospective participants were recruited through community‐based organisations (facilitated by [university research centre] staff) and snowball sampling. Self‐defined worker roles included community health workers, *promotoras*, and housing and labour rights advocates. All study procedures were approved by the University of California‐Merced's institutional review board, and each participant received a $50 gift card to honour their time.

The most common job title among participants was ‘*promotora’* (40%), followed by other community‐based outreach workers (32%) and those affiliated with a clinic network, community organisers or other specific roles such as legal aid outreach (27%).^1^ Interviews ranged from 30 to 89 min, with a median length of 49 min, and 46% of participants were interviewed in Spanish. Although we did not ask about participants’ gender, based on how participants referred to themselves, approximately 90% identified as women. Importantly, 28% of participants reported carrying out their work on a volunteer basis at some point during the pandemic—often because their legal status prevented them from obtaining U.S. work authorisation. Nearly half of the participants (46.5%) had been in their current role before the pandemic, approximately one‐third came into their role once the pandemic had started (32.5%), and it was unclear in approximately 21% of interviews exactly when the participant began their specific outreach work. This lack of clarity was often related to the fact that participants took on multiple overlapping roles, including volunteer and paid work, in multiple community‐related activities over time.

During interviews, we asked how the pandemic, as well as changing public benefits policies, shaped participants’ work in their communities. Bilingual research team members interviewed participants in their preferred language, and team members collaborated to code audio transcripts in their original language using Dedoose. A team of four researchers, including the faculty principal investigator (PI) and three students, met weekly at the early stages of analysis to discuss initial coding and identify emerging themes. Five process‐oriented themes emerged from the coded data: ‘identifying with community’, ‘problem solving’, ‘identifying worker‐related challenges’, ‘identifying community‐related challenges’ and ‘describing outreach work context’. Analysing specific codes related to these process‐oriented categories revealed participants’ experiential and interpretive expressions of their work within the context of a deadly virus, as well as how this work fit within a broader political economy of care work as morally—rather than materially—valued.

## 3 Findings

We organised Baehr’s seven ‘community of fate’ criteria into two broad categories: (1) the self‐evident aspects of the pandemic as *crisis* (danger recognition, trial, material/organisational resources and social rituals) and (2) the more implicit aspects of *care in community* (moral density, closure and axis of convergence). Regarding the first category, there was no doubt that COVID‐19 constituted an immediate danger to their own lives and the lives of family and community members and presented sustained trials that went beyond the initial shock of COVID’s arrival. These dangers and trials were only partially mitigated through the emergence of new COVID‐specific resources and social rituals such as masking and distancing. Because such factors are more self‐evident and require less elaboration, in the first empirical section, we focus instead on addressing the second category: *care in community*. We then expand our lens from the micro‐ to the macro‐level to understand the power dynamics between care work and the exacerbated care penalty that workers have experienced in the wake of the COVID‐19 pandemic.

### 3.1 Community Outreach Workers as a ‘Community of Fate’: Moral Density, Closure and Axis of Convergence

We begin with a focus on *closure*—a key aspect of Central Valley outreach workers’ pandemic experience that differed in several ways from the types of social distancing and temporary stay‐at‐home orders most people faced during the pandemic. Compared with other scholarship on pandemic communities of fate, which involved strict quarantine protocols for care workers, *closure* in the Central Valley functioned almost paradoxically as the impossibility of quarantine rather than a small‐scale closure in response to a contagious hazard. For example, the notion of closure as a feeling that compels people to ‘stand their ground’ because there is ‘little, if any, chance of flight or individual escape from the common lot…’ (Baehr 2005, 186) reflected the experiences of Central Valley immigrant communities—particularly community outreach workers—as a unique community of fate. However, the sense of strict quarantine (within a medical facility, for example) did not. Although the cases of the 2003 SARS outbreak in Hong Kong (Baehr 2005) and critical care workers in England during early COVID‐19 (Montgomery et al. 2021) reflected more narrowly bounded communities of fate who ‘[experienced] their predicament as collective exile’ (Baehr 2005, 186), Central Valley outreach workers’ experiences of pandemic closure were paradoxically more universal *and* more alienating.

Rather than collective exile, although the whole world seemed to close down in response to the unprecedented threat of COVID‐19, immigrant communities in California’s Central Valley had to continue their essential work as others retreated into the relative safety of their homes. The outreach workers we spoke with were especially essential and therefore disproportionately exposed to risk of infection, illness and death. Through their social position, they experienced a paradoxical kind of reverse closure, an alienation borne of being unable to retreat from the threat and instead facing it head‐on despite the risks. This social and occupational distinction means that outreach workers, by definition, could not be closed off from others. Their work emphasised the dual nature of communities of fate, one that shares a common destiny but is also shaped by the choices that individuals make to exercise constrained agency under uncertain conditions.

Outreach workers’ unique sense of closure came through most powerfully when participants described how they showed up in places where essential‐worker families and neighbours were present to share resources and information. Community organiser Alicia (all names are pseudonyms) emphasised the distinction—and importance—of community outreach workers’ commitment to being out in the field, saying, ‘…we have been present, our doors have been kept open, and we have gone where others have not’. Farmworker rights advocate Miguel similarly described the constant effort required to sustain this outreach work. ‘If you don’t go out [and] speak… to the community, it’s really difficult for people to come to us, to seek help ….’ Likewise, as *promotora* Sofía reflected:

We worked more than anybody because for us there was practically no pandemic, there wasn’t that closure. On the contrary, we had to be out front with the [COVID] tests. … we were risking ourselves to possible contagion… There we were, at the worst of the pandemic we always were working outside. Always, always, always.

Sofía’s remarks underscore the paradoxical nature of ‘closure’ for community outreach workers, who experienced alienation through their inability to self‐sequester, rather than through social isolation itself.

This unique sense of closure—paradoxically alienating and embedding—often sprang from participants’ sense of interconnectedness with their community and understanding that their own fate was inexorably entangled with those around them. This *moral density* of feeling called to intervene often extended from similar work in their professional lives or took the form of adapted skills, knowledge and networks from their personal lives (especially in schools and churches) to do COVID outreach work. They articulated a sense that *their community* (often expressed in co‐ethnic terms) shared a common predicament and interest beyond their own personal sphere. Moreover, this expression of moral density went beyond the abstract feeling that everyone in the world was somehow impacted by the pandemic. As those around them fell ill, and as they fell ill themselves, this social connection was material and immediate.

Community health worker Elena described how she saw herself and her family in her broader community. ‘To some of us, it is personal’, she remarked. ‘I like to think of it as that’s somebody’s family member. If it was my family member, I would want somebody to do the impossible to help them’. *Promotora* Carmen expressed a similar sense of interconnectedness as she described falling seriously ill with COVID and losing her father to the virus. ‘I don’t want another grandchild […] to lose their grandparents just because of ignorance and for not vaccinating their parents’, she said. Such remarks emphasised how many participants perceived their broader communities as composed of families like theirs and therefore felt a personal stake in their health and well‐being.

This sense of interconnectedness and shared fate also stemmed from collective histories of immigration and agricultural work. For example, the aforementioned community organiser Alicia described growing up in a family of farmworkers and experiencing income, housing and food insecurity first‐hand. ‘I made a promise to myself, coming from an immigrant family, that I wanted to work and do what I can to contribute to and work in communities where I identify myself’, Alicia explained, ‘coming as an immigrant, as a Latina, as a Mexicana’. Likewise, *promotora* Sandra described a feeling of embeddedness and solidarity with the farmworker community. ‘[More] than anything, our base [of our outreach work] is in support for farmworkers, and I'm also a farmworker’. *Promotora* Mayra, who worked with an organisation similar to Sandra's, echoed such sentiments, saying:

[Because] we are also immigrants, people identify with us, they feel confident to listen to us… It’s not the same thing to offer vaccines and share information on social media, but to do it personally. … it’s a labour of persuasion, that closeness to the people, that familiarity …

For community outreach workers in the Central Valley, this ‘moral density’—a sense of inexorable interconnectedness and shared fate—was often expressed as a common language and feeling of social cohesion. Baehr refers to such expressions as an ‘axis of convergence’ for communities of fate, emphasising that co‐ethnic ties often create its foundation (Baehr 2005, 189). Participants frequently articulated this through the metaphor of having their ‘heart’ in the vital work they performed. Because so many of the workers saw their families reflected in the broader communities they served, they also described their labour in terms of those intimate ties.

For example, Sara recalled telling her organisation that ‘we work for the community, …whenever we go out and we serve the community, we don't see people, we see our grandfathers, our grandmothers, our brothers and sisters’. Likewise, *promotora* Fernanda described seeing her mother, who died from COVID, in the community members she reached through her work. She brought in dozens of people to vaccine clinics:

In honour of my mother, I’m going to invite and [try to] convince as many people as I can, … because I don’t want so many people to keep dying. … I would say, ‘If there had been a vaccine, my mom wouldn’t have died. I’m going to take away all my pain by doing this with people, … and in this way I’ll have a little bit of satisfaction and peace in my heart.

Others also spoke of the heart when describing their initial and continued motivation for outreach work during the pandemic. As housing‐focused outreach worker Bianca explained, ‘All I can say is that community work you have to love it to be able to do it. If you don't love what you do for the community or for your people, then you're in the wrong job’. Alicia also described the work as rooted in a ‘tremendous love for community’ that often involved ‘tremendous sacrifice’. ‘It's not definitely for the money or anything like that’, she added emphatically. ‘It's tremendous love for community that moves us to do this kind of work’.

Collectively, participants' expressions of various aspects of a community of fate during the COVID‐19 pandemic illustrate how they made meaning of their efforts to facilitate health and well‐being resources on behalf of immigrant communities in the Central Valley—even in the face of low (or no) wages and ‘tremendous’ personal sacrifice. They articulated not only deep personal motivations and a sense of shared fate, but also unique risks and trials they confronted as a precarious, albeit essential, workforce. Although these challenges evolved somewhat over the course of the pandemic, there was a general sense that these emotionally rewarding efforts also exacted a mental and material toll on workers already occupying socially disadvantaged positions. In the following section, we explore this tension in terms of a ‘care penalty’ exacerbated by pandemic circumstances.

### 3.2 ‘Now We're Nothing but Volunteers’: Community of Fate and the Care Penalty

When asked, ‘How did you come to work here?’, participants often revealed a deep connection to community and a desire to become ‘a voice’ on its behalf. This sense of vocation, reflecting the *moral density* and *axis of convergence* discussed earlier, created a tension between work from the heart and work for which remuneration reflected participants' skill, expertise and danger exposure. Here, we first examine the micro‐level meaning‐making through which participants interpreted their work as a fated endeavour bound to their multifaceted roles in their families and communities. We then take a macro‐level perspective to examine how such evocations of fate may exacerbate a care penalty for already undervalued community outreach workers under pandemic conditions.

Whether they had worked professionally in community outreach before the pandemic or joined frontline outreach efforts only after it began, many described a long personal history of community service beyond the family—especially in schools and churches. This created a natural path towards pandemic community outreach work. For example, *promotora* Sofía explained that she ‘already had the soul of a *promotora*’ well before the pandemic, having long volunteered in the school system. Likewise, *promotora* Fernanda explained that although she always had taken care of others at home and through church service, the pandemic suddenly transformed her role from volunteer to professional.

Beyond the sense that service was a natural extension of a lifetime of care work in immigrant communities (particularly among women), many participants evoked a sense of calling or vocation when describing how they became outreach workers. For some, like *promotora* Estela, it was a literal call that spurred her into action. She had trained as a certified *promotora* 4 years earlier but ‘didn't feel the need to go out’. ‘But then, when they called us up, I said, ‘Here I am. I'm ready. Let's go’. Similarly, Estela's colleague Erica explained that years before the pandemic, a nun had called her to the role, saying, ‘I like you as a *promotora*’. Others connected their life experience in their communities with a positive sense of mission or calling. Community organiser Ana described growing up in a farmworker community that was active in farmworker rights movements as a ‘way of life’. ‘It's not a job’, she explained. ‘It's more of … a vocation…’ Likewise, community outreach worker Miguel, who focused on farmworkers' labour rights, said the pandemic made him feel fated to put more effort [*ganas*] into his work. ‘Somehow someone put us here’, he mused. ‘I don't know if it was destiny or if it was God, I don't know who it was that brought us here to be the voice of the community…’

Conceptualising Central Valley community outreach workers as a ‘community of fate’ during the COVID‐19 pandemic helps convey how participants interpreted their work as a labour of love emanating from the heart or spirit. Yet, recalling how Weber (2002) conceptualised the transformation of ‘vocation’ under capitalism (from a calling to enter the seclusion of spiritual life into potentially any worldly work done earnestly in God's honour), our analysis of participants' reflections reveals a tension between vocation as service from the heart or soul and professional *work* for which they are adequately compensated. Therefore, although an interpretive level of analysis is important for understanding how participants came to undertake such high‐risk work under daunting conditions and how they made meaning of that work, it is less helpful for understanding the material consequences of the gendered, racialised and legally stratified aspects of this community of fate. Here, we integrate a macro‐level perspective that considers the role of power relations and inequality in promoting a symbolic valuation of community outreach as materially undervalued care work. We argue that these vital workers faced an exacerbated ‘care penalty’ under pandemic conditions, which rhetorically praised their actions without sustained material investment. That their work was a vocation, and that it was essential, justified this devaluation—both among outreach workers with moral conviction but few other economic opportunities and those who might praise, but not pay, them as they would other uniquely skilled professionals.

Moreover, because the pandemic disproportionately impacted racialised immigrant communities, it exacerbated the existing care penalty for Central Valley outreach workers who were simultaneously caring for *and members of* communities the federal government explicitly excluded from pandemic relief resources. U.S. welfare and immigration laws have long excluded millions of noncitizens from government‐sponsored social benefits, and the first Trump administration expanded these exclusions just as the pandemic proliferated (Tafolla et al. 2024). Additional policies that further restricted social benefits and pandemic‐specific resources by legal status limited the extent to which outreach workers could support immigrant communities—including their own families. Many had to do more with less during the most demanding periods of the pandemic, thus compounding the existing care penalty of their gendered and racialised labour via legal status stratifications.

For many, the misalignment among benevolent motivations; their effectiveness in connecting with so‐called ‘hard‐to‐reach’, highly COVID‐impacted immigrant communities; and lack of material acknowledgement reflected workers' social position as immigrant (sometimes undocumented) women. For example, *promotora* Teresa had long felt called to outreach work but faced barriers to paid outreach work due to her legal status. ‘I've always wanted to be helping like that’, she explained, ‘but a lot of times you also can't help because there are places that … ask that you have papers [work authorisation] ….’ Teresa had felt ‘inspired’ to do farmworker outreach because her mother had faced many challenges in the fields, but she was discouraged from pursuing such work because she could only do it as a volunteer—especially after state labour law reclassified contract workers as ‘employees’ (Van Natta et al. 2023).^2^ ‘…[H]onestly you have to think about the fact that you're going to be helping for free’, Teresa remarked candidly, ‘they're not going to be paying you’. Yet, even when she was not compensated for her work, Teresa expressed a desire to continue. ‘Even so, we're going to keep working hours on this because it's our work. We do it from our heart…’

*Promotora* Julia's remarks echoed this tension. She briefly received compensation for the outreach work she was doing, but her legal status made this a precarious situation. ‘Now we're nothing but volunteers’, Julia explained. Yet, Julia wanted the opportunity for remunerated work because ‘we also want to have a motivation to keep doing [this work]’. She elaborated on this tension, saying that even though ‘they live to do [this outreach work]’ and ‘don't expect anything in return’, it would be nice to have income to offset the costs they incurred through their work (e.g., transportation costs).

Julia's colleague Carmen emphasised the difficulty of doing meaningful but undervalued work, especially as an undocumented outreach worker. She received valuable training as a *promotora* but lamented frequently having to work without sufficient pay (or any pay) or adequate resources. ‘Because at the beginning, we were purely volunteers, we did volunteer work’, she explained. ‘Later they began to pay us. … For me, when they begin to pay you some money for work that you're doing, where they have it, that's fair’. However, due to changing policies and the uneven nature of temporary grants—especially during the pandemic—*promotoras* could seldom count on stable work. ‘Suddenly feeling like they want you to keep doing the work, but not to pay you, that is a big challenge’, Carmen concluded.

*Promotoras* Veronica and Laura also reflected on this tension between dedication to meaningful work and feeling undervalued. Veronica only received wages for one day per month, despite doing outreach work multiple days per week. Laura echoed this experience, saying, ‘The majority of our work is not paid, we're volunteers…[but] I wanted to do something for my community’. Laura articulated how this commitment to community put her in a precarious position, not shared evenly among all community workers. ‘This work that we're doing deserves pay. It's not just free’, she asserted. ‘Yes, I've got a good heart…but … I think they should offer a wage to the *promotoras*’. Laura emphasised she was not necessarily asking for full‐time work, but her volunteer work during the pandemic opened her eyes to the fact that other people (in more longstanding roles in formal organisations and agencies) were receiving wages for the same work. ‘Because I learned…during all this time that they don't work for free’, she added. ‘They get grants, they get resources. …[This organisation I work for] has never paid me. …Really, the ones who do this work are us’.

*Promotora* Erica likewise described intense frustration over the hypocrisy of organisations relying on undocumented women's volunteer work to enhance paid staff's effectiveness. ‘They don't want to [pay me], but they want me as a volunteer’, she remarked. ‘I tell them we also eat, wear clothing, pay rent. And it's not fair that we do the work, and they don't give us anything. They tell us they can't pay us, but they want us as volunteers every day’. Erica added that from her perspective, paid staff used undocumented women to ‘open doors’ in wary communities, leveraging trusted local women like her in exchange for a little bit of ‘coffee and bread’ rather than real wages.

The reliance on immigrant workers—especially undocumented women—revealed how longstanding and emerging legal status stratifications in immigrant communities intensified outreach workers' care penalty. Well before the pandemic, legal status exclusions prevented many documented and undocumented immigrants from working in the formal sector (with formal labour protections), accessing health care and receiving state support such as unemployment insurance and financial assistance. Some of these services fell under the ‘public charge’ rule, which penalised immigrants seeking lawful entrance and residence in the country whom the government considered a ‘burden’ (Tafolla et al. 2024). In 2019, the Trump administration expanded the types of services considered under the rule, and pandemic relief resources explicitly excluded undocumented immigrants *and* households that included at least one undocumented person (Tafolla et al. 2024).

Several participants highlighted this policy shift as a major challenge to pandemic outreach work. *Promotora* Carolina, for example, described undocumented immigrants as ‘the most affected by the pandemic’. ‘Why?’ she mused. ‘Because they were essential workers, they had to work because it all is a chain’. This chain involved limited savings due to low wages and being unable to quit their ‘essential’ jobs. Because of this, Carolina observed that ‘…when the pandemic arrived, everyone lost control’. When asked what would make their jobs easier, several participants called for the removal of legal status barriers to aid. ‘Because you’re undocumented, we cannot help you’, said community outreach worker and DACA recipient Alex.^3^ Likewise, outreach worker Carla emphasised that her organisation’s funds only covered eligible citizens or lawful residents.

Many outreach workers experienced this intensification of legal status exclusions both professionally and personally, reinforcing pandemic *closure* in racialised, low‐income immigrant communities in a rural region with few economic opportunities. As the expanded public charge rule and pandemic aid restrictions complicated outreach work without increasing wages (or—sometimes—even providing any wages), participants often faced the same challenges as the communities they served: low‐ or no‐wage work without health or unemployment benefits. *Promotora* Sofía described this period as ‘traumatising’, recounting that despite her professional knowledge, her husband became gravely ill with COVID and could not access the care he needed due to his legal status. Sofía connected this tragedy to the government's broader abandonment of immigrant communities during the pandemic—including lack of funding for outreach workers. ‘Because to get grants to keep working has been very, very difficult’, she explained. ‘Even knowing that they [government officials] see our work, see our labour, see everything, it's not easy to get grants. They put in many restrictions, precisely for the type of population that we have [immigrants]…’

These examples underscore how women from immigrant communities who were best equipped to connect Central Valley immigrants to vital resources faced a compounded care penalty because of their unique positionality. Several factors led to this undervaluation, including community organisations' inability to formally contract employees who lacked work authorisation—even when they were the best suited for these positions—and the limited duration of ‘emergency’ COVID‐19 public funding at the state and county level (Van Natta et al. 2023). Gloria, a nonprofit programme manager, emphasised the need for creative solutions to compensate undocumented workers engaged in vital outreach work. ‘Not to circumvent the law’, she added, ‘but to allow people to sell their labour as if they were a business or a cooperative. […] [T]hat's the type of advocacy that we need in community to improve our wellness’. Many others expressed the need for public agencies and policymakers to recognise that the pandemic did not spontaneously end when agencies decided to kerb funding and that its consequences would endure and require sustained investment rather than retrenchment.

## 4 Discussion

As the COVID‐19 pandemic descended on California's Central Valley, frontline outreach workers often felt called to mobilise their knowledge, networks and heart to bring vital resources to the communities in which they lived and worked. Their unique combination of personal motivation, connection to community and professional training—alongside the sociopolitical and economic closure that bound rural immigrant communities to ‘essential’ low‐wage labour and pandemic relief exclusion—constituted a ‘community of fate’ during an unprecedented, inescapable crisis. Although the vastness of the pandemic made it ubiquitous, the community outreach workers we spoke with represented a distinctive community of fate bound by disproportionate risk and exceptional engagement. Unlike communities with more privileges and resources, participants lived and worked in communities that could not retreat into relative isolation to await ‘normal’ life. Like many of their family members and neighbours, these outreach workers were ‘essential’ and sensed that whatever befell their community would also befall them.

The community outreach workers we spoke with leveraged their skills and social positions to fight pandemic disruptions despite the frequent frustration and marginalisation they experienced because of that unique combination of training and social location. The disparity between the physical and emotional intensity of these efforts and their material valuation revealed how the pandemic exacerbated a fundamental ‘care penalty’ latent in outreach work. Although they gave their all for those they cared about deeply, the personal sacrifices this demanded during a protracted disaster such as COVID intensified the urgency of addressing longstanding questions about equity and fair labour conditions. Ultimately, the very motivations that allowed participants to push through crisis as a pandemic ‘community of fate’ may also justify devalued labour, which threatened their lives and demanded urgent attention.

Our study aligns with earlier sociological insights on the ‘care penalty’ and deepens our understanding of how additional structural conditions—such as legal status stratification and a pandemic—can compound that penalty. We found that Central Valley outreach workers often described their labour as work from the heart *and* work that was undervalued. At the same time that the pandemic drew these workers together as a community of fate, it also exacerbated the devaluation of their heartfelt labour by demanding that they do more with less—more uncompensated or poorly remunerated time spent working under more dangerous conditions and fewer resources to draw upon given their own and/or their communities’ legal status exclusions.

Sociologists have identified key mechanisms whereby societies come to devalue racialised and gendered care work, including its association with undervalued members of society (i.e., women, especially women of colour), care work as a public good that cannot be priced, the intrinsic sanctity of care work as altruistic labour emanating ‘from the heart’ and the commodification of emotional labour (e.g., England 2005). By examining these dynamics empirically through the pandemic lens, we have illuminated how care workers in California’s Central Valley made meaning of their work during a time of intense danger, uncertainty and socioeconomic exclusion. We also demonstrated how the community of fate that motivated their labour and gave it that meaning suffered from a similarly intensified devaluation that other ‘essential workers’ in their communities faced during the pandemic. Unlike those working in other ‘essential’ roles, however, the notion of outreach work as a labour of love for one’s community helped obscure and justify the devaluation of that work.

Our study also echoes past research emphasising the ways that governments and nongovernmental organisations rhetorically celebrate community outreach worker programmes for ‘empowering’ a primarily female workforce while, in fact, granting them little meaningful access to financial remuneration or political decision‐making. By training low‐income women to intervene where the state is most absent, such workers come to occupy the lowest position within healthcare bureaucracies, where they were regarded as ‘disposable labour to be disciplined’ rather than agents of advocacy and policy change (Closser et al. 2019, 300). Although the pandemic revealed how ‘essential’ outreach workers are to managing a public health crisis, it has not sparked concomitant investment in centring such workers in long‐term workforce development or policymaking conversations (Van Natta et al. 2023). This is especially alarming when one considers that within California, as well as globally, rising wealth inequality is creating a greater need for redistributive policies to support deliberate safety‐net investment (Qureshi 2023).

Future scholarship should examine the nuanced pathways whereby the pandemic may have compounded the undervaluation of outreach work as care work in immigrant communities, including through racialised, gendered and legally stratified processes. As pandemics continue to disrupt the health and livelihoods of immigrant communities in California's Central Valley and similar communities throughout the United States, our findings highlight the need to adequately remunerate and safeguard the well‐being of community outreach workers. Unfortunately, these workers' sense of calling has rationalised the valuation of their vital contributions in symbolic rather than material terms. We suggest an unmet need to prioritise workforce development investments that value the unique experiences, knowledge and skills of these workers to the same extent that their ‘essential’ labour has been praised at the rhetorical level throughout the pandemic. Relying on the precarious, low‐wage and sometimes volunteer labour of women in immigrant communities exacerbates an enduring gendered and racialised ‘care penalty’ for such workers and makes clear the long‐term need for a more sustainable and equitable community outreach workforce that will endure beyond the pandemic's lingering challenges.

## Author Contributions

**Meredith Van Natta:** conceptualization (lead), data curation (equal), formal analysis (lead), funding acquisition (lead), investigation (lead), methodology (lead), project administration (lead), resources (lead), supervision (lead), writing – original draft (lead), writing – review and editing (lead). **Andrés Arias:** conceptualization (supporting), data curation (lead), formal analysis (supporting), project administration (supporting), writing – review and editing (supporting). **Maria Andrea Escobar:** conceptualization (supporting), data curation (lead), formal analysis (supporting), writing – review and editing (supporting).

## Ethics Statement

This study received ethics approval from the University of California, Merced, Institutional Review Board.

## Consent

No patients were included in this study.

## Conflicts of Interest

The authors declare no conflicts of interest.

## Permission to Reproduce Material From Other Sources

No reproduced material from other sources is included in this article.

## Data Availability

To protect the security of study participants, the data for this study are not available to the public.

## Endnotes

^1^
The term *promotora* (or *promotor* in the masculine form) refers to a ‘trusted person that empowers their peers through education and connections to health and social resources’ who often works in ‘Latino/x and Spanish‐speaking communities’ (California Health Care Foundation 2024). Many *promotoras/es* complete formal certification through outreach‐worker training programmes (Gutiérrez et al. 2021).

^2^
See California Assembly Bill 5 (2019) on the worker status of employees and independent contractors, which classified certain workers as ‘employees’ that may otherwise be misclassified as ‘contractors’. This impacted undocumented workers who may have been authorised to work through self‐employment on a contract basis but were not authorised to be classified as ‘employees’. Some undocumented interview participants described becoming unauthorised for wage labour and moving (back) into volunteer roles because of this policy shift, a challenge that temporary COVID‐related grant funding compounded.

^3^
DACA, or ‘Deferred Action for Childhood Arrivals’, refers to a precarious (frequently challenged) federal programme instituted in 2012 that provides temporary relief from deportation for qualifying immigrants who arrived in the United States without legal authorisation as children.
